# Supplementary material for: Covalent-bonding-induced strong phonon scattering in the atomically thin WSe2 layer
Source: Sci Rep. 2019 May 20;9:7612. doi: 10.1038/s41598-019-44091-9 (PMC6527611; doi:10.1038/s41598-019-44091-9)
Supplement: Supplementary file 1 — Supporting Information [file 41598_2019_44091_MOESM1_ESM.docx]

SUPPORTING INFORMATION

Covalent-bonding-induced strong phonon scattering in the atomically thin WSe_2_ layer

Young-Gwan Choi^1#^, Do-Gyeom Jeong^1^, H. I. Ju^1^, C. J. Roh^1^, Geonhwa Kim^1^, Bongjin Simon Mun^1^, Tae Yun Kim^2^, Sang-Woo Kim^2^, and J. S. Lee^1*^

^1^Department of Physics and Photon Science, School of Physics and Chemistry, Gwangju Institute of Science and Technology, Gwangju 61005, South Korea

^2^School of Materials Science and Engineering, Sungkyunkwan University, Suwon 16419, South Korea

**1. Sensitivity analysis of time-domain thermoreflectance measurements**

From the time-domain thermoreflectance measurement (TDTR), we obtained the thermal boundary conductance G_n_ of the metal-substrate interface with the mono- (n=1) and bi-layered (n=2) WSe_2_ in between. Here, we analyze the sensitivity of each physical parameter involved with the measurement, and demonstrate that G_n_ is the most sensitive parameter among considered. The sensitivity (S_p_) of a certain parameter p is defined as S_p_=d(ln(V_in_/V_out_)/d(lnp)). It should be noted that all parameters other than the parameter of interest are fixed as values presented in the main
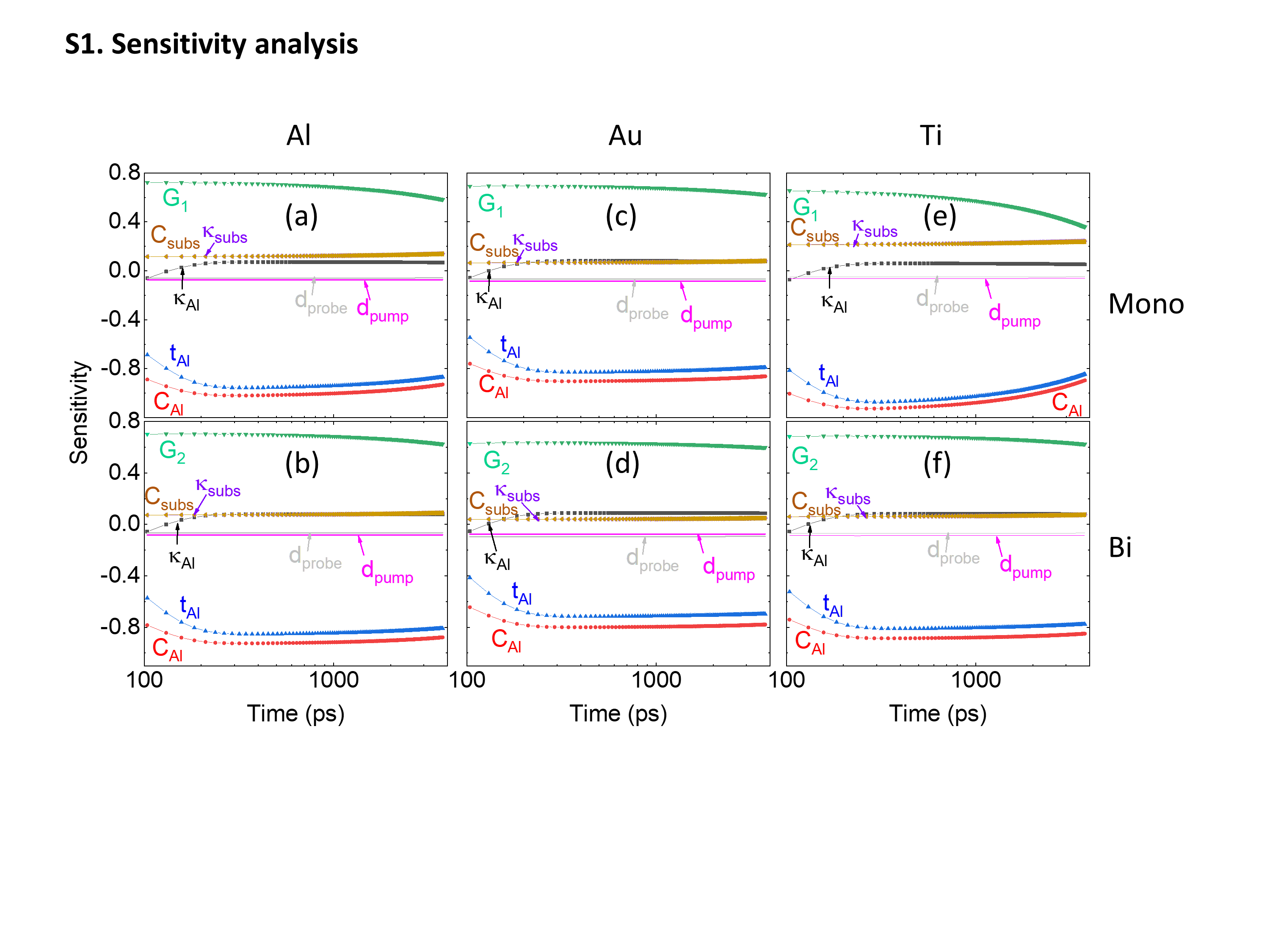
text. For example, G_n_ is given as (G_1_, G_2_)=(22.2, 16.3), (15.3, 11.3) and (36.2, 14.5), in a unit of MW/m^2^K, for the Al-, Au-, and Ti-contact, respectively. A sapphire thermal conductivity (κ_subs_) is obtained by fitting the TDTR results as 28.7±2.5 W/mK. And, some parameters are referred to the literature values, such as the heat capacity of aluminum (C_Al_)^S1^ and sapphire (C_subs_)^S2^.

**Fig. S1.** Sensitivity simulations for WSe_2_ flakes on sapphire substrate with different metal contact condition, aluminum (a,b), gold (c,d), and titanium (e,f) for mono- and bi-layered WSe_2_. Here, G is the thermal boundary conductance, C_subs_ is the heat capacity of sapphire, κ_subs_ is the thermal conductivity of sapphire, t_Al_ is the thickness of metal layer, κ_Al_ is thermal conductivity of aluminum, C_Al_ is the heat capacity of aluminum, d_pump_ and d_probe_ are sizes of pump and probe beams.

Figure S1 summarizes the delay-time-dependent sensitivity to several physical parameters, i.e., the size of the pump (d_pump_) and the probe (d_probe_) beam, C_Al_, C_subs_, κ_subs_, the Al thermal conductivity (κ_Al_), the Al thickness (t_Al_), and G_n_. Among them, G_n_ has the highest sensitivity larger than 0.6 in the entire time-domain investigated. Hence we can successfully decouple each G_n_ value from other physical parameters, and determine them with a high reliability.

**2. Uniformity of WSe_2_ flakes**


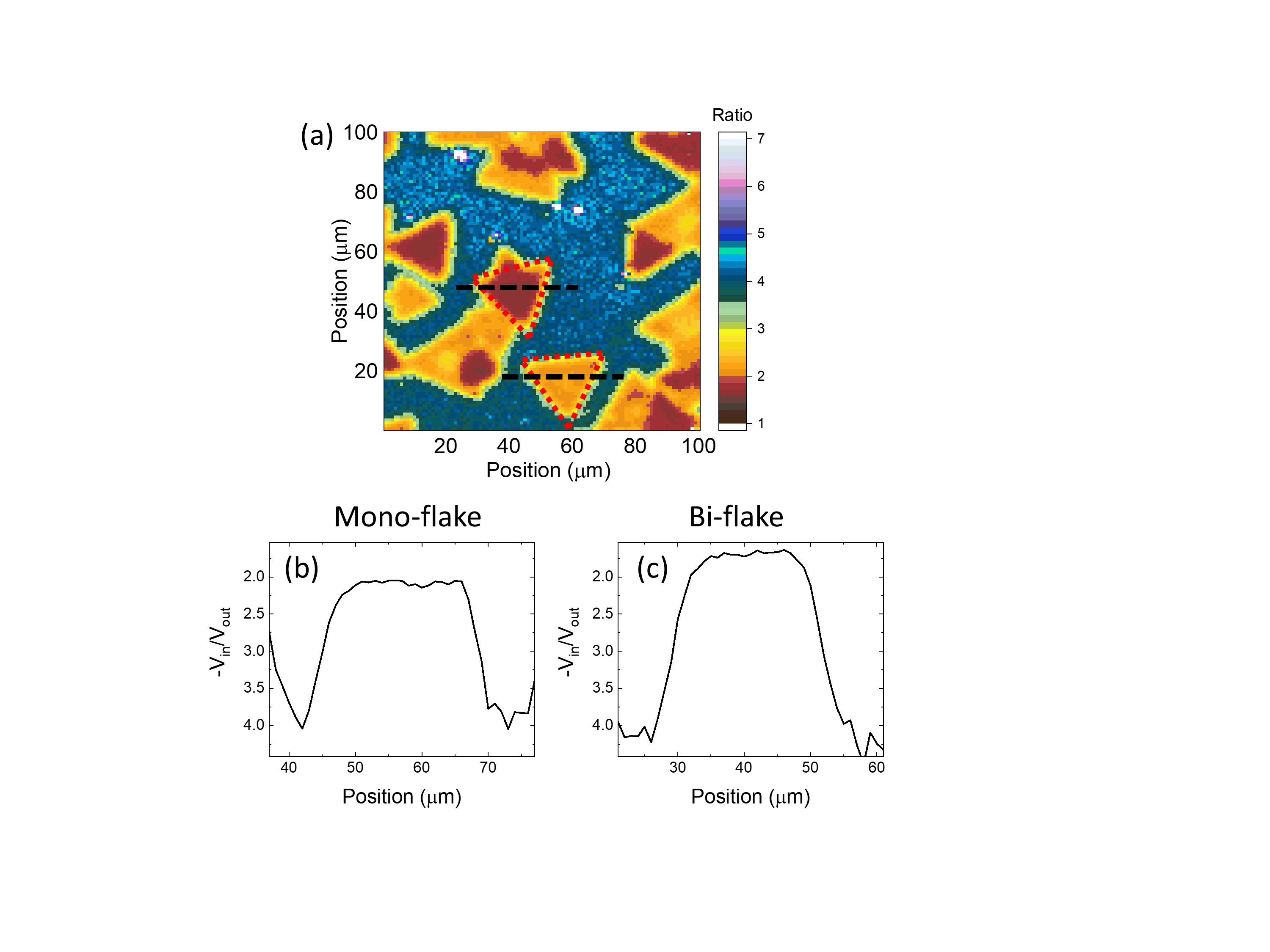
Although the TDTR mapping results clearly exhibit homogeneous responses of each WSe_2_ flake, we confirm the uniformity of the flake in more detail by examining the line profile results. Figures S2(b,c) display the line profiles of the -V_in_/V_out_ signal for the mono- and bi-flake WSe_2_ of which two-dimensional mapping result is shown in S2(a). Near the boundary, a rising/declining slope is clearly discernible over a distance of about 5 μm which is in good agreement with the pump/probe beam size. Inside of the flakes, we can find a uniform distribution of the signal over about 15 μm which is wider than the beam size. This confirms that each flake has a homogeneous distribution of the thermal property, and its thermal parameter represented by G_n_ can be extracted without any ambiguity for each layer number n.

**Fig. S2**. Time-domain thermoreflectance mapping data (a), and its line profile for (b) mono- and (c) bi-flake WSe_2_. Ratio signal is roughly proportional to the thermal boundary conductance. In Fig. S2 (a), yellow triangles correspond to mono-flake WSe_2_ and brown triangles correspond to bi-flake WSe_2_.

**3. X-ray photoemission spectroscopy analysis**


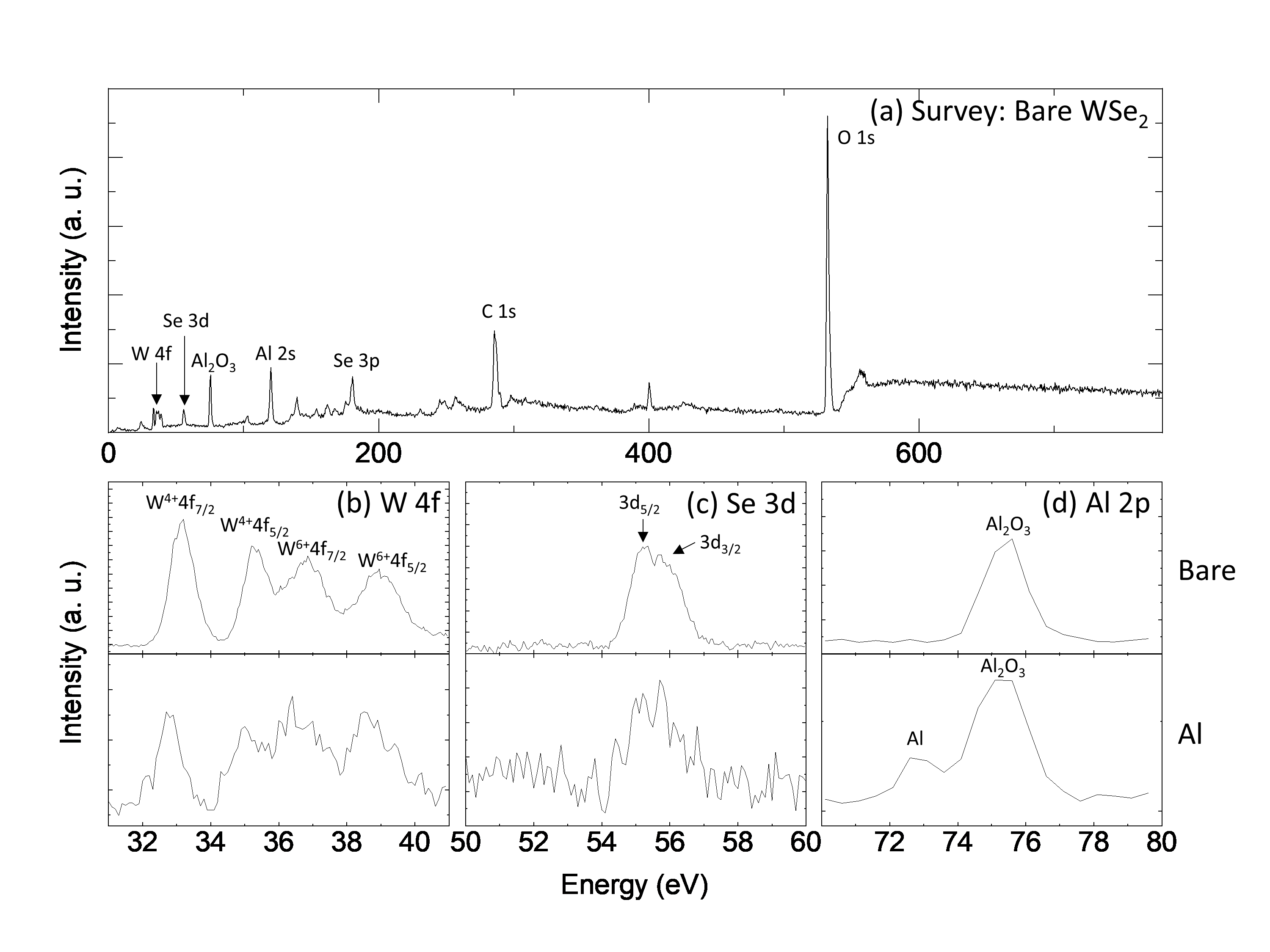
Here we provide x-ray photoemission spectroscopy (XPS) results for bare and Al-deposited WSe_2_ specimens in more detail. Figure S3a shows the survey signal of the bare WSe_2_ which is in good agreement with reference data^S3^. Figures S3(b-d) display the atom-specific XPS spectra for the bare WSe_2_ and Al-deposited WSe_2_, shown in the upper and lower row, respectively. For the W 4f peaks, there is a large difference between the bare and Al-deposited WSe_2_ which is discussed already in the main text. For the Se 3d peaks, 3d_5/2_ and 3d_3/2_ responses are well characterized in the bare WSe_2_ flake. However, the corresponding responses become much weaker for the Al-deposited WSe_2_ flake so that they are not discussed in regard to the bonding relation between metal and WSe_2_. Finally, Al 2p peaks are well discernible for both bare and Al-deposited WSe_2_ flakes. Interestingly, the Al 2p state of the metallic state is clearly seen for the Al-deposited WSe_2_ flake^S4^. This means that even if the Al layer might be oxidized at its surface, the bare Al layer should remain under the oxidized Al layer. Therefore, our assumption about the Al-WSe_2_ contact instead of Al_2_O_3_-WSe_2_ should be secured. Although we expect the largest modulation of the WSe_2_ surface when it is in contact with Ti, we cannot get enough XPS signals due to a strong scattering of photo-electrons in the Ti layer.

**Fig. S3**. X-ray photoemission spectroscopy signals of bare WSe_2_ and Al-deposited WSe_2_.

**References**

S1. Shackelford, J. F. and Alexander, W. Thermal Properties of Materials. In Materials Science and Engineering Handbook; CRC Press LLC: Boca Raton, FL; p 398 (2001).

S2. Archer, D. G. Thermodynamic Properties of Synthetic Sapphire (α-Al_2_O_3_), Standard Reference Material 720 and the Effect of Temperature-Scale Differences on Thermodynamic Properties. J. Phys. Chem. Ref. Data **22**, 1441−1453 (1993).

S3. Tao, L. Meng, F. Zhao, S. Song, Y. Yu, J. Wang, X. Liu, Z. Wang, Y. Li, B. Wang, Y. & Sui, Y. Experimental and theoretical evidence for the ferromagnetic edge in WSe_2_ nanosheets. Nanoscale, **9**, 4898 (2017).

S4. Strohmeier, B. R. An ESCA Method for Determining the Oxide Thickness on Aluminum Alloys. SURFACE AND INTERFACE ANALYSIS, **15**, 51-56 (1990).
